# Evidence on the prevalence, incidence, mortality and trends of human papilloma virus-associated cancers in sub-Saharan Africa: systematic scoping review

**DOI:** 10.1186/s12885-019-5781-3

**Published:** 2019-06-11

**Authors:** Kabelo M. B. Lekoane, Desmond Kuupiel, Tivani P. Mashamba-Thompson, Themba G. Ginindza

**Affiliations:** 1 Discipline of Public Health Medicine, School of Nursing and Public Health, University of KwaZulu-Natal, 2nd Floor George Campbell Building, Howard College Campus, Durban, 4001 South Africa; 2 Research for Sustainable Development, Sunyani, Ghana

**Keywords:** Human papilloma virus, Human papilloma virus -related cancer, Incidence, Prevalence, Mortality, Trends, SSA

## Abstract

**Background:**

Human papilloma virus (HPV) associated cervical cancer remains a global concern particular, in Sub-Saharan Africa (SSA) where the impact is felt most. Evidence show that many other cancers such as vaginal, anal, oropharyngeal, penile are because of persistent infection with *HPV* especially, high-risk types.

**Aim:**

We mapped evidence on the incidence, prevalence, mortality, and the trends of human papillomavirus-related cancers in SSA.

**Methods:**

A comprehensive literature search was conducted from several databases including PubMed, Google scholar, Science Direct, and CINAHL and MEDLINE via EBSCOhost as well as World Health Organization website for grey literature. Studies reporting HPV-related cancers in SSA outcomes including prevalence, incidence, mortality, and trends were included in this study. The risk of bias of the included studies were assessed using the mixed methods appraisal tool version 2011. We employed PRISMA (preferred reporting items for systematic reviews and meta-analyses) to report the search results. Thematic analysis used to reveal the emerging themes from the included studies.

**Results:**

Seventy-four (74) studies were retrieved at full article screening, eight of them (six reviews, and two quantitative study) were eligible for data extraction. The degree of agreement between the two independent reviewers following full article screening, was 86.49% agreement versus 64.57% likely by chance which constituted moderate to significant agreement (Kappa statistic = 0.62, *p*-value< 0.05). Of the eight included studies, four (50%) studies generalized about SSA with no country of interest; two (25%) studies were conducted in Nigeria; one (12.5%) reported about Uganda, Zambia, Guinea, Malawi Tanzania, Mali, Mozambique, Zimbabwe; and one (12.5%) reported about Ethiopia, Senegal, Zimbabwe and Uganda. These eight included studies reported evidence on more than one outcome of interest. Four studies reported about the prevalence of HPV-related cancers, seven studies reported about the incidence, four studies reported about mortality, and four studies reported about the trends of HPV-related cancers.

**Conclusion:**

This study observation highlighted a gap of knowledge regarding the epidemiological data on the recent HPV prevalence in SSA, which will have a potential impact in determining the distribution of HPV on different body sites (cervix, penis, vagina, vulva, anus and oropharynx). Ongoing research projects are recommended in SSA to enhance the value of HPV, and HPV-associated cancers epidemiological data to inform strategies or/and policies on prevention, diagnosis, and treatment of HPV-related conditions.

## Background

Human Papilloma Virus (HPV) is a small non-enclosed deoxyribonucleic acid virus that mostly infect the cells of the epithelium [1]. Human papilloma virus is known as one of the common causes of sexually transmitted infections (STI’s) worldwide [2]. Globally, more than 170 HPV genotypes have been identified so far [3], and 15 of them (16, 18, 31, 33, 35, 39, 45, 51, 52, 56, 58, 59, 68, 73, and 82) are highly recognized as high-risk HPV types (hr-HPV) and are intricate in HPV-related cancers development [4, 5]. HPV strains 16 and 18 are highly prevalent in Africa and they are noted to be the major cause of over 65% cervical cancer cases in the SSA [6, 7]. Over 95% of cervical cancers are said to be associated with hr-HPV infection, as well as about 75–90% anal cancers and a significant percentage of vaginal, penile, vulvar cancers, and oropharyngeal [8].

Globally, over 4% of all types of cancer cases, almost 2% of cancers in high-income countries (HICs), and nearly 8% in low-income countries (LICs) are attributable to hr-HPV [9]. The highest prevalence of cervical HPV infection is reported in Sub-Saharan Africa (SSA), with Guinea (48%) and Mozambique (41%) been the most report [10, 11]. According to World Health Organization (WHO), by 2030 cervical cancer will be responsible for 443,000 deaths of women worldwide [12]. Ninety-eight percentage of the predicted deaths will occur in LICs, with SSA having the highest number of deaths [11]. Body of evidence on HPV-related anogenital cancers is increasing, according to de Martel C et al., worldwide estimation of new cases yearly for vaginal cancer is 13,000, 27,000 for vulvar cancer and 27,000 for anal cancer respectively [13]. Penile cancer incidence rates are higher in LICs than in HICs. It is estimated that penile cancers account for about 22,000 (9.5%) cancer cases worldwide in Africa, South America, and Asia [14].

However, progress has been made to prevent the HPV infection through development of the three FDA approved HPV vaccines; Gardasil®, Gardasil® 9, and Cervarix® and they protect against HPV disease causing types [15, 16]. These vaccines provide protection in women aged 9–26 years against HPV 6, 11, 16 and 18-associated cervical cancer, and others such as anogenital cancers (vaginal and vulvar), adenocarcinoma in situ, cervical intraepithelial neoplasia (CIN), vulvar intraepithelial neoplasia and vaginal intraepithelial neoplasia and genital warts [17]. The bivalent vaccine also provide protection in women aged 10–25 years against HPV 16 and 18- associated cervical cancer, adenocarcinoma in situ and CIN grades 1 [18]. We therefore, aimed to map evidence on the incidence, prevalence, mortality as well as trends of HPV-related cancers in SSA. It is envisaged that the findings of this study will, help to strengthen the implementation of preventative strategies and improve the preventive and control and treatment, programs to reduce the burden and mortality of HPV-related cancers in SSA. It is also anticipated that the results of this study will benefit public health research by identifying gaps on current studies for future research.

## Methods

This study forms part of the larger study titled; “Mapping evidence on the distribution of Human Papillomavirus-related cancers in sub-Saharan Africa: scoping review”. This study is registered in PROSPERO under registration number CRD42017062403. A thorough description of the methods used in this study can be found in the published protocol [19].

### Search strategy

We conducted a thorough literature search in the following databases: PubMed, Science Direct, Google scholar, and CINAHL and MEDLINE with full text via EBSCOhost platform for published studies. We also search WHO website for grey literature. The database search occurred from March 2017 to July 2017. Titles of the eligible studies were searched by using the following keywords combination in the databases “HPV-related cancers”, “prevalence”, “incidence”, “mortality”, “trends”, and “Sub-Saharan Africa”. Keywords during the search were separated by the usage of Boolean terms AND/OR. Language and date restrictions were not applied on the search. There was inclusion of Medical Subjects Headings (MeSH) terms in the search. Additional eligible articles identified by reviewing reference sections of articles.

### Eligibility criteria

#### Inclusion criteria

- Evidence of study conducted in Sub-Saharan Africa
- Study that included individuals with HPV-related cancers
- Evidence that focus on prevalence, incidence, mortality and trends of HPV-related cancers

#### Exclusion criteria

- Studies that do not focus on HPV-related cancers
- Studies not focusing on humans
- Qualitative studies were excluded

Firstly, one reviewer searched for the titles of the eligible studies from the databases based on the eligibility criteria. Duplicates studies were removed. Secondly, two reviewers screened all retrieved abstracts and they were evaluated for eligibility using the inclusion criteria. Studies that included humans’ beings and reported at least one outcome of this study were selected. Where the reviewer was uncertain with the eligibility of study population, intervention, outcome and study setting were eligible the article was included in the next stage for screening. Agreement between the reviewers about potentially relevant studies was reached by consent and the full text obtained for screening. Thirdly, full article screening was done by two independent reviewers and a third investigator engaged to address disagreements between reviewers. We used Cohen’s kappa coefficient (κ) statistic in Stata version 13 to calculate degree of agreement between reviewers.

### Charting data

Data from the included studies was extracted using a formulated data form. For all studies, information on the author and date, study setting, outcome (prevalence, incidence, mortality and trends), age, percentage of males/females, gender, study designs were extracted.

### Summary and collating

Thematic analysis was performed to show evidence on the prevalence, incidence, mortality as well as the HPV-associated cancer trends in SSA from the included studies. The results from the included studies were summarised and manually coded and categories or themes as follows:

- Prevalence
- Incidence
- Mortality
- Trends

### Quality assessment

The mixed methods appraisal tool version 2011 was used to assess the methodological quality of the included studies [21]. Of the eight included studies, two of them were assessed to determine their quality. Two reviewers independently performed the quality assessment and the results were communicated. The following domains were used to score the risk of bias of the included studies: research questions clarity, the research question confidence assessment, suitability of the data sources collected; statistical analysis appropriateness to address the research question. Additional domains included are as follows: the design and allocated concealment clearly described, complete data outcome and the withdrawal, recruiting of participants, the suitability of measurements and comparable participants, applicable sample and representative of the population, applicable design and mixing of methods and researchers’ suitably considered method. An overall quality score percentage of the included studies was calculated, and scores interpreted as ≤50% low quality, 51–75% average quality, and 76–100% high quality. The included studies overall quality percentage score was calculated, and scores were interpreted as high quality (100%).

## Results

**Fig. 1:**
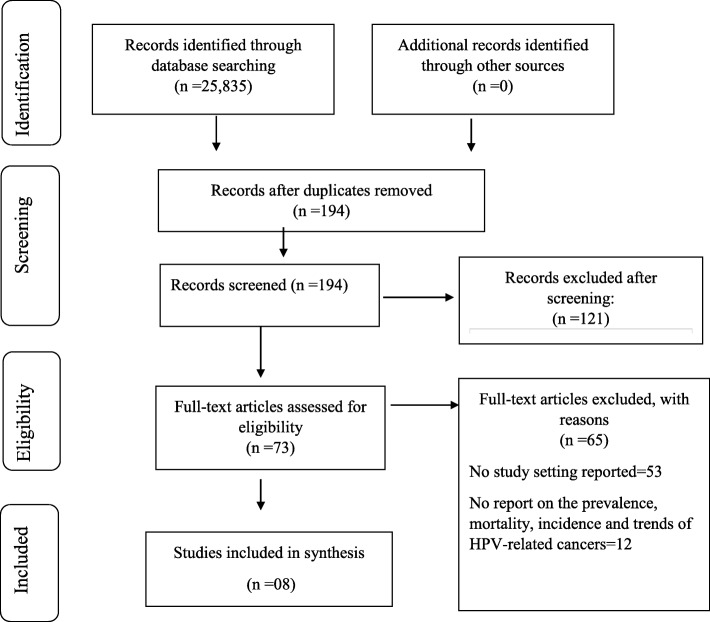
PRISMA chart showing literature search and selection of studies

We obtained 25,835 articles from all databases during the initial search. About 306 articles qualified for abstract screening, after exclusion of duplicates about 194 different articles remained. Of those, about 73 articles were recognized for full article screening. Based on the review of the full article screening, 65 articles were excluded in this study. Then eight articles were deemed qualified for inclusion in data withdrawal. Applying our inclusion criteria, the number remained at eight (Fig. 1).

Following full article screening, there was 86.49% agreement versus 64.57% expected by chance between the two independent reviewers which constituted of moderate to substantial consensus (Cohen’s kappa = 0.62, calculated probability of < 0.05). In addition, the chi-square statistic by McNemar’s implies that ratios of yes/no by reviewer is not a statistically significant difference with *p*-value > 0.05.

From the eligible 194 studies considered for screening, an overall of 121 studies were unable to meet the eligible criteria and were deemed unsuitable for this study. Meanwhile, a sum total of 73 studies were selected for full article screening, 65 of them did not have important data to be used in this study. A total number of 53 articles did not report evidence on the study setting of interest which is SSA [22–74] and 12 did not report anything about the prevalence, incidence, mortality, and trends of HPV-related cancers [75–86].

### The elements of the included studies

Included studies elements are presented on Table 1. We included research work that focused on individuals with HPV-linked cancers, conducted within SSA focusing on the prevalence, incidence, mortality and trends of cancers linked to HPV. The studies comprised of 75% (6/8) of review articles [10, 87–91], and 25% (2/8) of quantitative study [92, 93].

**Table 1:** Elements of the included studies

Author and date	Study population			Study setting	Study design	Outcome reported
	gender and age	number or population percentage	type			
Louie et al., 2009 [90]	Men, women	Not specified	Not specified	Sub-Saharan Africa (SSA), Zimbabwe, Uganda, Ethiopia Senegal	Review	Prevalence, incidence
De Vuyst et al., 2013 [10]	Women	Not specified	Not specified	SSA, Guinea, Zambia, Tanzania, Malawi, Mozambique, Zimbabwe, Uganda, Mali	Review	Prevalence, incidence, trends
Bosch et al., 2013 [87]	Women	Not specified	Not specified	SSA	Review	Prevalence, incidence, mortality, trends
Arbyn et al.,2011 [88]	Women	Not specified	Not specified	SSA	Review	Incidence, mortality
Forman et al., 2012 [91]	Women	Not specified	Not specified	SSA	Review	Incidence, mortality
Jedy et al.,2016 [92]	Men, women	Not specified	Not specified	Nigeria	Descriptive cross- sectional study	Prevalence, incidence, trends
Oga et al., 2016 [93]	Men, Women	Not specified	Not specified	Nigeria	Retrospective study	Prevalence
De Sanjose et al., 2014 [89]	Women	Not specified	Not specified	SSA	Review	Incidence, trends

**Fig. 2:**
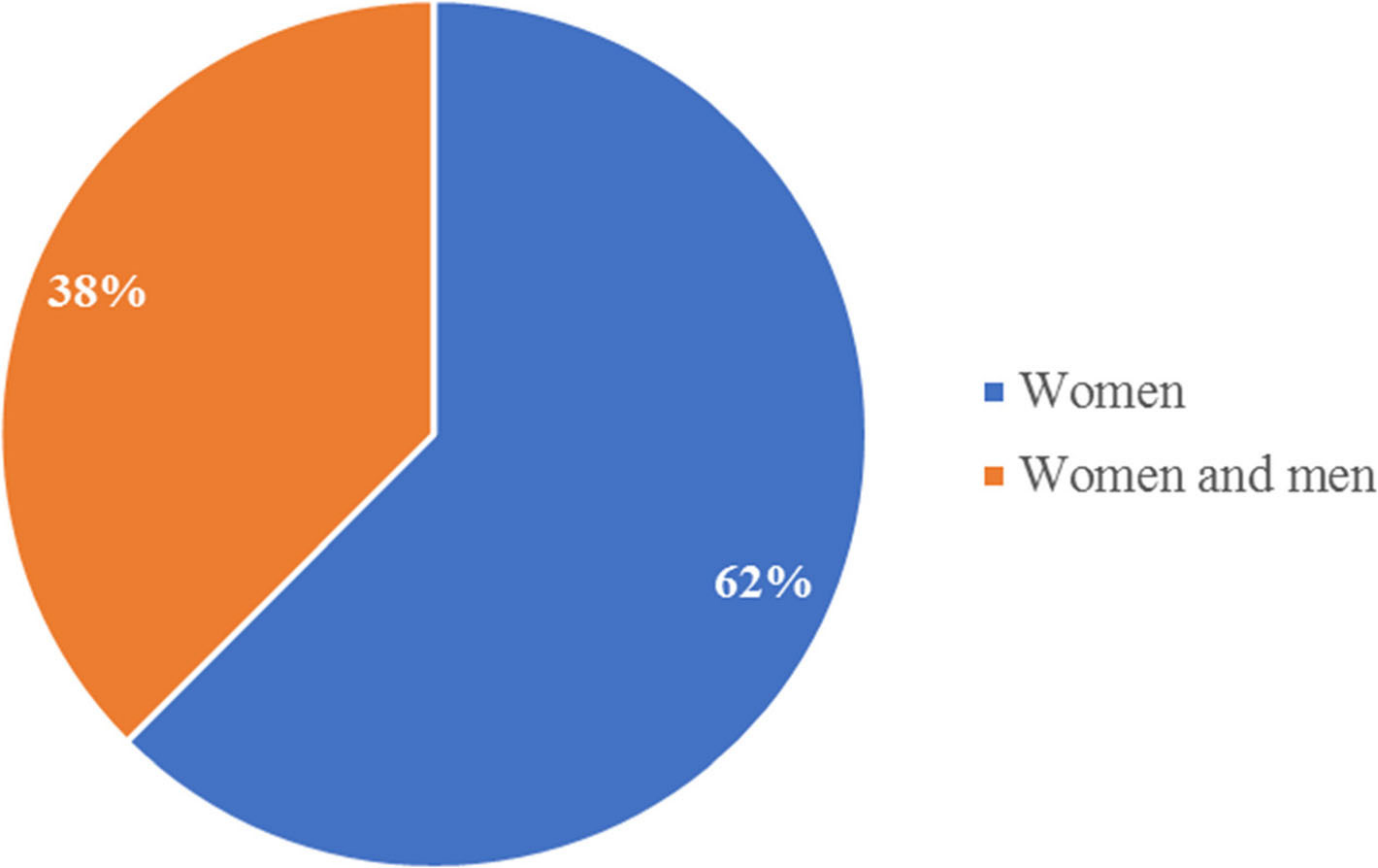
Study population of the included studies

These studies were conducted and reviewed in different study setting, approximately 50% (4/8) studies generalized about SSA, with no country of interest [87–89, 91], 25% (2/8) study was conducted in Nigeria [92, 93], and one (1/8) multi-country study reported about Uganda, Zambia, Guinea, Malawi Tanzania, Mali, Mozambique, Zimbabwe [10]. Another one (1/8) multi-country study was also reported about Ethiopia, Senegal, Zimbabwe and Uganda [90]. It was not mentioned in all the studies whether they were conducted in urban or rural setting. Of the included studies, 62.5% (5/8) studies reported about women [10, 87–89, 91], and 37.5% (3/8) reported about both men and women [90, 92, 93] as illustrated in Fig. 2.

From the eight included studies, four studies reported about HPV-related cancers prevalence [87, 90, 92, 93], seven studies reported about the incidence [10, 87–92], three studies reported about mortality [10, 87, 91], and four studies reported about HPV-related cancers trends [10, 87, 89, 92]. Age of the population and the sample size of participants were not mentioned in the included studies.

The Mixed Methods Appraisal Tool (MMAT) – Version 2011 was used to assess the methodological quality of two primary studies, from the eight included studies [27]. The studies scored 100% [92, 93] and were considered to be of high methodological quality. The evidence showed overall minimal risk of bias.

### Study findings

The following subtopics were presented to analyse the data from the included studies: prevalence; incidence; mortality; and trends of cancers linked to HPV.

#### Prevalence

Out of the eight included studies, four studies reported on the prevalence cancers associated with HPV infection [87, 90, 92, 93]. Amongst women with normal cytology in SSA, it’s been found that HPV infection prevalence is topmost at 24% as compared to worldwide prevalence of around 11–12% [87]. The overall estimated widespread presence of HPV 16/18 infection linked to invasive cervical cancer (ICC) cases in SSA is 69.2% which is coherent with the estimation of 70% worldwide [90]. HPV 16 and 18 prevalence in ICC cases were found to be 43.7% in Senegal and 90.2% in Ethiopia [90].

The severity of lesions among women with CIN grade 3 and ICC has been reported to be about 90% which is directly proportional to the prevalence of women with cytologic cervical pathology [87]. Prevalence of human immunodeficiency virus (HIV) in SSA is linked to several HPV-related cancers, supported by a cancer registry in Nigeria, which reported a two-fold increase of cervical cancers in people living with HIV (PLWH) [92]. A retrospective study by Oga and colleagues [93],demonstrated a minimal widespread presence of hr-HPV linked to head and neck cancers amongst Nigerians [93]. This current study based on this finding, observed that HPV attributable cervical cancer prevalence is increasing in SSA.

#### Incidence

Seven of the included studies reported about the incidence of HPV-related cancers [10, 87–92]. Increased incidence rates of cervical cancer were noted in SSA in the entire world [10] and it ranks second after breast cancer in all SSA countries and its sub-regions [10]. Additionally, not less than four-fold higher incidence rates of cervical cancer are reported in LIC’s countries predominantly SSA in comparison with very HIC’s [87, 91]. According to Jedy et al., (2016) cervical cancer has shown to be commonly linked to HPV [92]. This was supported by similar incidence findings reported from West Africa at 29.3 per 100,000 and Central Africa at 28 per 100,000 and much higher occurrences reported in Southern Africa which amount to 38.2 per 100,000 and Eastern Africa that sum to 42.7 per 100,000 of cervical cancer incidence [92].

Worldwide, incidence rates of ICC were topmost at > 50/100,000 women which are noted at the following countries; Malawi,Guinea, Tanzania, Zambia, and Mozambique [10]. Comparable results reported in a review by Louie et al., demonstrated an estimated overall age standardized incidence rate (ASR) of 31 per 100,000 women of ICC cases [90]. Global penile cancer incidence rates correlate with those of cervical cancer is estimated to be 26,300 cases annually [88]. The penile cancer incidence is noted to be higher in LMIC’s, accounting to about 10% of penile cancers in certain regions of Africa [88].

Globally, an increasing incidence of anal cancer by 2% per year has been observed for the past three decades [92]. Annually, 27,000 new cases of anal cancer were diagnosed with an average worldwide incidence rate that sum to 1 per 100,000 [89]. However, in Uganda low occurrence rates of anal cancer have been reported at 0.2 per 100,000 in men and women and < 0.1 per 100,000 in both Zimbabwean men and women [92]. Study performed in Nigeria, showed low incidence of oropharyngeal cancers attributable to HPV as low in Nigerian men and women [92]. There is rise in penile cancer incidence in some parts of Africa and low incidence rates of anal and oropharyngeal cancers observed in other countries within SSA.

#### Mortality

Three of the included studies reported about HPV-related mortality [10, 87, 91]. Mortality rates is reported to be at least four-fold higher in LMICs mainly in SSA [87, 91]. Taking everything into account, within SSA the age-standardized incidence and mortality rates (ASIR and ASMR) of ICC are high at 31.7 and 22.5 per 100,000 women with comparison to 9.1 and 3.1, respectively, in HICs [10]. In the SSA region, when estimated ICC is responsible for 1,474,208 deaths in women aged 15 years or more [10]. Most of the studies showed that mortality in SSA due to HPV-related cancers is higher as compared to HICs.

#### Trends

Out of the eight included studies, about four of them reported about the trends of HPV-related cancers [10, 87, 89, 92]. Review by de Vuyst et al., demonstrated from selected registries trends of cervical cancer incidence has been observed [10]. The ten-year trends for Mali between 1987 and 1989 and 1994–1996 were increased from 23 to 36/100,000 over time, between 1991 and 1993 and 1998–2002 in Kyadondo, Uganda was 41 to 46/100,000 and between 1990 and 1992 and 1998–2002 in Zimbabwe a decrease was observed from 65 to 48/100,000 [10]. In the nineties, both Uganda and Zimbabwe experienced extremely elevated widespread presence of HIV among the general population, but opposite cervical cancer trends were showed over time, however with increased ASIR of 52/100,000 women for the period 2002–2006 [10]. In LMIC’s there is limited information for trends of HPV linked oropharyngeal cancers [92], again possible underreporting for time trends for anogenital cancers is observed in LMIC’s [89], associated with haphazard variations inherent to the small numbers involved [87, 89]. The studies showed in SSA, time trends for cervical cancer is increasing and data on trends for other anogenital cancers is limited.

## Discussion

This systematic scoping review provided evidenced-based knowledge on the prevalence, incidence, mortality and trends of cervical, anal, penile and head and neck cancers in SSA. The findings revealed a lack of research in different geographic locations and poor participation among men. Our results demonstrated that there is limited evidence on the recent studies that explores the prevalence, incidence, mortality and trends of HPV-related cancers in SSA. Additionally, our results presented a scarcity of studies focusing on other HPV-related cancers other than cervical cancer. In our observation, evidence of cervical cancer attributable to HPV dominates vaginal, anal, vulvar and head and neck cancers, the cancers are probably underreported as the evidence on their burden is limited. Unfortunately, data were insufficient for describing HPV-attributable vulvar or vaginal cancers. This study observation highlight a gap of knowledge on the epidemiological data on the recent HPV prevalence in SSA, which will have a potential impact in determining the distribution of HPV on different body sites (cervix, penis, vagina, vulva, anus and oropharynx).

This study revealed that incidence and mortality ratios of cervical cancer in SSA are higher than other parts of the world [10]. Similarly, the review by Arbyn et al., 2012, in 2008, mortality to incidence ratio amounted to 52% worldwide, with an estimation of 275,000 deaths of women from cervical cancer whereby approximately 88% occurred in LMICs [88]. This study shows low incidence rate of anal cancer reported among men and women in Uganda and Zimbabwe [92]. In contrast, a study which was conducted in the United States of America (USA) showed increase incidence rates of anal cancer in both males and females [94, 95]. This study also demonstrates low incidence of oropharyngeal cancer among Nigerians meanwhile study findings in the USA reported increased incidence rate of oropharyngeal cancer particularly, in men [96, 97]. In this study, the penile cancer incidence is noted to be higher in LMICs, accounting for 10% of penile cancers in some parts of Africa [88]. Meanwhile an increasing incidence of vulvar, vaginal and penile cancers have been reported in Denmark [98] and Netherlands [99]. However, incidence rates for penile cancers and vaginal cancers have declined during the period 1973–2006 in the US [100, 101]. Worldwide, head and neck cancers are noted as the eighth most common cancer with approximately 650,000 new cases and 350,000 deaths reported each year [102]. According to GLOBOCAN 2012 report, worldwide Age Standardized Incidence Rate (ASR) of head and neck cancers were found to be 10.7 per 100,000 in the United States and 9.0 per 100,000 population on the African continent [103].

All inclusive ASIR and ASMR of ICC are high in SSA amounting at 31.7 and 22.5 per 100,000 women as compared with 9.1 and 3.1, respectively, in more HICs [10], with less than two per 100,000 women reported in Western Europe, Australia and New Zealand [104]. This current study revealed an increase of cervical cancer ASIR between the period 2002–2006 [10], however this may be due to possible underreporting of time trends for anogenital cancers in LMICs [89]. Comparing with European cervical cancer in Scotland, the ASRs ranged from 7.7 to 11.8 per 100,000 women between the 5-year periods of 1972–1976 and 2007–2011 [105]. However, constant temporal rates of penile cancer are noted in Scotland and England [103]. There is declining trend of smoking associated with low incidence of head and neck squamous cell carcinoma in developing world [106]. However, there is limited data in Africa checking the review from cancer registry in SA, increase in oropharyngeal cancers among “coloured” South Africans from 1992 to 2001 was noted [106].

### Strengths and limitations of the study

The methodology used in this study, systematic scoping reviews, allows for extensive literature search, with no date and language restriction. This study was also not limited to any age or population group. A systematic scoping review guided by Arksey and O’Malley’s framework was used and it was the best method to map the evidence on cancers linked to HPV [20]. The PEO (population, exposure, and outcomes) framework was used during selection of studies as it was the appropriate heuristic to use based on the framed research question. Additionally, the results of the scoping review were presented following PRISMA recommendation, which ensured complete and transparent reporting. The MMAT tool version 2011 was used to assess the methodological quality and assess the risk of bias of the included studies.

We acknowledge the limitation in this scoping review. This systematic scoping study was limited to quantitative studies due to the use of PEO nomenclature. The exclusion of qualitative studies might have resulted in omission of some valuable data. Providing relevant solutions in this study has been a challenge as the literature of different cancer sites, especially for cancer of anal, vaginal, vulvar, and head and neck on this topic is limited. It is also possible that researches on the prevalence, incidence, mortality and trends of HPV-related cancer outcomes may exist under different terminologies not considered in this study. However, we included MeSH terms in the search to address this limitation.

### Implications for research

This study shows limited recent evidence on the epidemiological data of HPV-related cancers especially on anal cancer across all genders, vaginal cancer, vulvar cancer, head and neck cancers and penile cancer. Additionally, we recommend more future studies to be conducted at different countries within SSA and in rural areas. It also calls for recruitment of more males to participate in research. Ongoing studies to explore the incidence, prevalence and mortality of these cancers by country and cancer sites in SSA are recommended. Trends in ASIR in SSA are not favorable, therefore more studies to determine the time trends of HPV-related cancers are required.

### Implication of practice

This study revealed the need for policy makers in public health to take note on the prevalence of HPV in SSA, and development of HPV attributable cancers and to inform action or/and policies on preventing, diagnosing, and treating HPV-related conditions. The presence of low-cost technologies for testing HPV and inclusion of HPV vaccines should be made available in SSA. Better yet, after the first three-dose course of HPV vaccination in Rwanda has resulted in 93, 23% coverage which showed good response [107], therefore other countries should be motivated to expand their vaccination programmes. Well recorded cancer registry data to assess the burden of non-cervical cancers is recommended.

## Conclusion

This study enabled the authors to provide evidenced-based knowledge on the prevalence on the prevalence, incidence, mortality and trends of HPV associated cancers in countries within SSA. This review also highlighted a literature gap on recent epidemiological data on HPV prevalence in SSA, which may have a potential impact in determining the distribution of HPV on different body sites (cervix, penis, vagina, vulva, anus and oropharynx). Therefore, future research projects are recommended in SSA to enhance the value of data for consistent reporting on the epidemiology of HPV-related cancers.

## Abbreviations

ASIR: Age-standardized incidence rate
ASMR: Age standardized mortality rate
ASR: Age-standardized incidence
HDI: Human development index
HIV: Human immune-deficiency virus
HPV: Human papilloma virus
Hr-HPV: High-risk human papilloma virus
IARC: International agency for research on cancer
ICC: Invasive cervical cancer
LMIC: Low-and-middle-income countries
MMAT: Mixed methods appraisal tool
PLHIV: People living with HIV
SSA: Sub-Saharan Africa
STI: Sexually transmitted infections
WHO: World Health Organization
